# Quality of Life and Utility in Patients with Metastatic Soft Tissue and Bone Sarcoma: The Sarcoma Treatment and Burden of Illness in North America and Europe (SABINE) Study

**DOI:** 10.1155/2012/740279

**Published:** 2012-03-20

**Authors:** Peter Reichardt, Michael Leahy, Xavier Garcia del Muro, Stefano Ferrari, Javier Martin, Hans Gelderblom, Jingshu Wang, Arun Krishna, Jennifer Eriksson, Arthur Staddon, Jean-Yves Blay

**Affiliations:** ^1^Department of Hematology, Oncology and Palliative Care, Sarcoma Center Berlin-Brandenburg, HELIOS Klinikum Bad Saarow, 15526 Bad Saarow, Germany; ^2^Department of Medical Oncology, Christie Hospital, Wilmslow Road, Withington, Manchester M 204 BX, UK; ^3^Medical Oncology Unit, Institut Català d'Oncologia, Gran Via, s/n km 2,7, 08907 L'Hospitalet de Llobregat, Barcelona, Spain; ^4^Sezione di Chemioterapia dei Tumori dell'Apparato Locomotore, Istituto Ortopedico Rizzoli, Via Pupilli 1, 40136 Bologna, Italy; ^5^Hospital Son Dureta, Andrea Doria 55, 07014 Palma de Mallorca, Spain; ^6^Department of Clinical Oncology, Leiden University Medical Center (LUMC), Albinusdreef 2, 2333 ZA Leiden, The Netherlands; ^7^Health Economic Statistics, Merck Research Laboratories, Upper Gwynedd, PA 19454, USA; ^8^Global Health Outcomes, Merck Research Laboratories, Whitehouse Station, NJ 08889, USA; ^9^Ingenix Pharmaceutical Services AB, Optum Insight, Klarabergsviadukten 90, Hus D, 11164 Stockholm, Sweden; ^10^Pennsylvania Hematology Oncology Associates, 230 West Washington Square, 2nd Floor, Philadelphia, PA 19106, USA; ^11^Department of Medical Oncology, University Claude Bernard Lyon I, 43 Boulevard du 11 Novembre 1918, 69622 Villeurbanne cedex, Lyon, France

## Abstract

The aim of the study was to assess health-related quality of life (HRQoL) among metastatic soft tissue (mSTS) or bone sarcoma (mBS) patients who had attained a favourable response to chemotherapy. We employed the EORTC QLQ-C30, the 3-item Cancer-Related Symptoms Questionnaire, and the EQ-5D instrument. HRQoL was evaluated overall and by health state in 120 mSTS/mBS patients enrolled in the SABINE study across nine countries in Europe and North America. Utility was estimated from responses to the EQ-5D instrument using UK population-based weights. The mean EQ-5D utility score was 0.69 for the pooled patient sample with little variation across health states. However, patients with progressive disease reported a clinically significant lower utility (0.56). Among disease symptoms, pain and respiratory symptoms are common. This study showed that mSTS/mBS is associated with reduced HRQoL and utility among patients with metastatic disease.

## 1. Introduction

Sarcomas are rare cancers of connective tissue, such as bone, muscle, nerves, fatty tissue, and cartilage. Sarcomas fall into three broad categories, soft tissue (STS), primary bone cancers, and gastrointestinal stromal tumors (GISTs); there are, however, many subtypes of sarcoma within these categories. The incidence of STS in the US is 2.4 to 3.6 per 100,000 inhabitants per year depending on age and race, and the incidence in Europe is 3.2 per 100,000 per year [1, 2]. 39% of patients have advanced disease (defined as regional or metastatic) at their initial diagnosis, whereof 15% present with metastatic disease [1]. Among those presenting with localized disease, approximately 35% of patients progress to metastatic disease [3]. Treatment with chemotherapy is the standard route of care in patients with mSTS and mBS [4, 5].

The most common form of bone sarcoma, osteosarcoma and Ewing's sarcoma, the second most common form of bone sarcoma, are cancers that most commonly affect adolescents [6]. Malignant fibrous histiocytoma (MFH), a former classification dismantled among different subtypes, including leiomyosarcomas, myxofibrosarcomas, and liposarcoma are most commonly affecting middle-aged or elderly individuals. The most common sarcoma types are leiomyosarcoma representing approximately 20% of total STS cases, MFH (19%), and liposarcoma (10%) contributing to approximately 50% of cases [7]. Other types of STS subtypes include synovial sarcoma (approximately 2.7% of total STS cases), fibrosarcoma (6.7%), Kaposi sarcoma (12%), dermatofibrosarcoma (8%), and sarcoma not otherwise specified (12%).

In order to assess the impact of a new medical treatment on resource allocation, one needs a measure that incorporates both survival and quality of life. A quality-adjusted life year (QALY) is a generic outcome measure that is constructed by weighting the length of time spent in different disease states by a utility value (on a scale of 0 to 1, where 0 represents dead and 1 best imaginable health) corresponding to the quality of life associated with the level of health status [8].

Utility weights can be estimated with the EQ-5D which is a standardized, non-disease-specific instrument for use as a measure of health outcomes. With the EQ-5D instrument, patients can rate health states which are then converted into utilities using a scoring algorithm. Such scoring algorithms have been developed based on the preferences of the general population [9]. Furthermore, there are also cancer-specific quality of life elicitation methods. One commonly employed questionnaire is the EORTC QLQ-C30 questionnaire which is developed to assess quality of life in cancer patients by assessing patient's overall quality of life, functionality, and disease symptoms [10].

There is limited published literature on HRQoL in metastatic sarcoma patients. Poveda and colleagues (2005) used the EORTC QLQ-C30 in 23 patients with metastatic sarcoma, by administering the instrument before and after treatment with pegylated liposomal doxorubicin [11]. Quality of life did not seem to worsen during therapy. Systematic literature review indicated that there are no published utility values collected in patients with metastatic sarcoma.

A primary objective of the sarcoma treatment and burden of illness in North America and Europe (SABINE) study was to describe utility weights in metastatic sarcoma patients who have attained a complete response (CR), partial response (PR), or stable disease (SD) following chemotherapy. The three HRQoL instruments used were the EQ-5D, the EORTC QLQ-C30, and the 3-item Cancer-Related Symptoms Questionnaire (originally used in SUCCEED trial [12], a phase III clinical trial among advanced sarcoma patients). Secondary objectives were to explore quality of life according to predefined health states and to identify factors that predict quality of life.

## 2. Methods

The present study was a cross-sectional patient survey where data on quality of life in patients with metastatic soft tissue sarcoma (mSTS) and metastatic bone sarcoma (mBS) were collected. Eligible patients were recruited consecutively (the reason being not to influence patient selection) by their treating physician at a total of 25 study sites across nine countries: Canada, USA, Germany, France, Italy, The Netherlands, Spain, UK, and Sweden. The study was approved by the ethical committees in each respective country and patient informed consent was obtained.

Patients included were 18 years of age or older, had a confirmed diagnosis of metastatic soft tissue or bone sarcoma and had had a favourable response (CR, PR, or SD) according to the WHO or RECIST 1.0 criteria after any line of chemotherapy. The following soft tissue subtypes were included: leiomyosarcoma, liposarcoma, and undifferentiated pleomorphic sarcoma/malignant fibrous histiocytoma, and the following bone sarcoma subtypes were included: osteosarcoma and Ewing's sarcoma. These subtypes were selected to reduce the heterogeneity in the patient sample. Patients enrolled in the SUCCEED trial were excluded as this study was intended to evaluate health-related quality of life in sarcoma patients as they are being treated in actual clinical practice, and to avoid interference with the SUCCEED clinical trial. Patients were enrolled in the patient survey between December 2009 and March 2011.

Patients were recruited according to predefined sarcoma-related health states related to the patient's line of treatment and response status. A maximum of five patients per site were allowed to be enrolled in each health state. The predefined health states are presented in Table 1. Off-chemotherapy was defined as being longer than six weeks since last dose of chemotherapy given the persistence of toxicity (e.g., fatigue and mucositis) following completion of a chemotherapy regimen.

The physician or study nurse answered a short questionnaire with demographic and treatment and disease-related questions. Three health-related quality of life elicitation questionnaires: European Quality of Life Questionnaire (EQ-5D), EORTC QLQ-C30, and a 3-item Cancer-Related Symptoms Questionnaire (used in the SUCCEED clinical trial) were administered to the patients at the physician visit at one single point in time.

The EQ-5D self-assessment questionnaire provides two analysis variables: EQ-5D visual analog scale (VAS) and EQ-5D descriptive system. For both VAS and utility, a higher score indicates better health, and the questions address the patient's health status on the day of questionnaire completion. Patients with missing data on the EQ-5D questionnaire were excluded from the utility calculations. EQ VAS is a visual analogue scale that ranges from 0 (worst imaginable health state) to 100 (best imaginable health state). The EQ-5D descriptive system defines health in terms of five dimensions: mobility, self-care, usual activities, pain or discomfort, and anxiety or depression. Each dimension is divided into three levels that correspond to whether the respondent has (1) no problem, (2) some problem, or (3) extreme problems thus consisting of 243 possible combinations. To each combination of answers, a utility value, anchored at 0 (death) and 1 (full health) is assigned. However, there are health states considered worse than death, which is why negative utility values may occur. The utility value is in turn based on a set of preference weights (tariff) elicited from general population samples by some other method, for example, the time trade-off (TTO) method. The tariff used in this study were EQ-5D value weights based on a TTO study from a UK population [9], with a sensitivity analysis using a tariff from a US population [13].

The EORTC QLQ-C30 questionnaire includes 30 items of which 28 have four response levels (not at all, a little, quite a bit, and very much). The remaining two items are rated on a scale from 0 (very poor) to 7 (excellent). The instrument is composed of both multiitem scales and single-item measures including five functional scales (physical, role, emotional, cognitive, and social functioning), nine symptomatic scales, and a global health status scale representing the quality of life. 25 items are based on recall period of seven days. The five items that physical functioning is composed of have no explicit recall period. In this study, missing values were not imputed as missing values appeared nonrandom.

The 3-item Cancer-Related Symptoms Questionnaire consists of three questions to describe the existence and severity (mild, moderate, or severe) of three symptoms; pain, cough, and shortness of breath. This questionnaire was developed for and subsequently used in the SUCCEED trial [12].

Clinically relevant differences were assessed in quality of life between different health states using the EORTC QLQ-C30 HRQoL and utility values. For EORTC QLQ-C30 variables which range from 0 to 100, the clinically relevant differences were determined as a “little change” (5–10 points change in the score), a “moderate change” (10–120 points), and “very much change” (>20 points) [14]. For EQ-5D utilities, which range from −0.59 to 1, a minimally important difference is 0.074 units [15].

The parameters estimated from the EORTC QLQ-C30, the 3-item Cancer-Related Symptoms Questionnaire and the utility values were calculated as means along with their respective standard deviations. An analysis of covariance regression analysis (ANCOVA) [16] was done to assess predictors of quality of life with the EORTC QLQ-C30 global health status score as dependent variable. The following covariates were included in the full model.

Demographic variables:

- age at survey completion (continuous variable),
- gender,
- region (categorical variables: North America: Northern Europe, Southern Europe):
  - Northern Europe is defined as Sweden, Netherlands, UK, and Germany,
  - Southern Europe is defined as France, Italy, and Spain.

Disease-specific variables:

- health state (categorical variable, 1–5 as specified previously in Table 1),
- pain (categorical variable, as measured by the symptoms questionnaire),
- cough (categorical variable, as measured by the symptoms questionnaire),
- shortness of breath (categorical variable, as measured by the symptoms questionnaire),
- number of metastases (continuous variable),
- time since diagnosis (continuous variable).

The ANCOVA regression was initially run including all independent variables listed above (i.e., the full model). Then, statistically insignificant covariates were manually removed using backward selection criteria and the model was rerun until all covariates were significant at a 5% level. The final model specification was also checked for heteroskedasticity and normality of residuals.

Statistical significance was evaluated at the 5% level. All *P*-values reported are two-sided.

Analyses were performed using SAS 9.2. All analyses were exploratory. The reported *P*-values should be considered only for informative purposes and not for drawing any definitive conclusions.

## 3. Results

### 3.1. Patient Characteristics

Of the 120 sarcoma patients enrolled into the study, 99 were mSTS patients and 21 had mBS. Four of the 120 patients were considered not valid for final study analysis due to the following reasons: missing response status (*n* = 1), did not complete any patient questionnaire (*n* = 1), enrolled in SUCCEED clinical trial (*n* = 1), and was too young (16.9 years old at survey completion) (*n* = 1). The evaluable patient set was therefore 116 sarcoma patients.

Table 2 shows the demographic and disease characteristics of the patients. Of the 116 evaluable patients, 96 (82.8%) patients had mSTS and 20 (17.2%) had mBS. 68 (58.6%) patients were female. The mean age at metastatic sarcoma diagnosis was 49.5 years (SD = 17.1), with a range of 16.1 to 83.8 years. Extremity (35.3%) followed by retroperitoneum (16.4%) were the most frequently reported site of disease. Note that patients could have more than one metastasis. The mean number of sites of metastases per patient reported was 1.4. The most common site of metastasis was the lung (44.6%). Other sites of metastases were bone, lymph nodes, pleura, abdomen, retroperitoneum, pancreas, mediastinum, skeletal, kidney, pelvis, and skull. The majority of patients (62.9%) were receiving chemotherapy at the time of questionnaire completion.

### 3.2. Health Utilities

The EQ-5D utility values are given in Table 3. The mean utility score was 0.69 for the pooled patient sample. Patients with mBS (0.68) and patients with mSTS (0.69) had a similar mean utility score, and the difference between the EQ-5D utility scores for mSTS and mBS patients was not statistically significant (*P* = 0.93).

The mean VAS score (range 0–100) was reported to be 65 for the pooled patient sample, 65.8 for mSTS patients and 61.3 for mBS patients. The observed difference was not statistically significant (*P* = 0.52).

In addition, EQ-5D utility values by health state (HS) for the pooled patient sample, using the UK weights, are presented in Table 4. Patients on 1st-line chemotherapy had an average utility score of 0.72, while the results for 2nd and 3rd line or higher were 0.64 and 0.77, respectively. Results were similar for patients off chemotherapy, preprogression (0.77). Patients with progressive disease reported a lower mean utility (0.56). VAS scores were also calculated by health state. In contrast to the results by health state using utility values, the VAS scores decline with increasing lines of therapy.

The nonparametric Mann-Whitney test was used to assess any potential statistically significant differences in median utility values between the following health states (HS): HS1 (median = 0.76) versus HS2 (median = 0.73); HS1 versus HS3 (median = 0.75); HS2 versus HS3; HS4 (median = 0.66) versus HS5 (median = 0.76). The median utility was calculated for each respective comparative group. These comparisons were selected because they were deemed as clinically meaningful comparisons. A statistically significant difference was found between HS4, progressive disease, and HS5, off-chemotherapy pre-progression, (*P* = 0.003). There were no other statistically significant differences between the compared health states.

A sensitivity analysis was also performed using the US EQ-5D tariff. This resulted in a mean utility value of 0.76 (SD = 0.18) for the pooled patient sample and hence a difference in mean utility of 0.075 compared to the UK-based utility (mean = 0.69, SD = 0.26). This is mainly due to the UK tariff yielding negative values, while there were none for the US tariff based utilities as well as a higher median value for the US tariff utilities. The analysis for utility values by health state was therefore rerun. The conclusions of the utility by health state analysis using the US-based tariff remained the same (data not shown).

### 3.3. Quality of Life

QoL as measured by the EORTC QLQ-C30 global health status variable indicated a similar mean quality of life score in the mBS subsample (63.3) and the mSTS subgroup (62.0). These results are presented in Table 5. In addition, the EORTC QLQ-C30 functional scale (range 0–100) was scored for the patient's physical, role, emotional, cognitive, and social functioning. The lowest score (indicating poorer quality of life) was reported for role functioning (mean 60.6, SD = 33.6) and social functioning (mean 65.9, SD = 29.1).

For the EORTC QLQ-C30 symptoms scale (range 0–100), where a higher value indicates more symptoms, scoring was performed for the common cancer-related side effects fatigue, nausea and vomiting, pain, dyspnoea, insomnia, appetite loss, constipation, diarrhoea, and financial problems. The highest symptoms scale scores were reported for the fatigue score (mean 40.1, SD = 28.4) followed by insomnia (mean 28.9, SD = 29.9) and pain (mean 27.6, SD = 27.2). The results were consistent in both mSTS and mBS patients.

**Figure 1:**
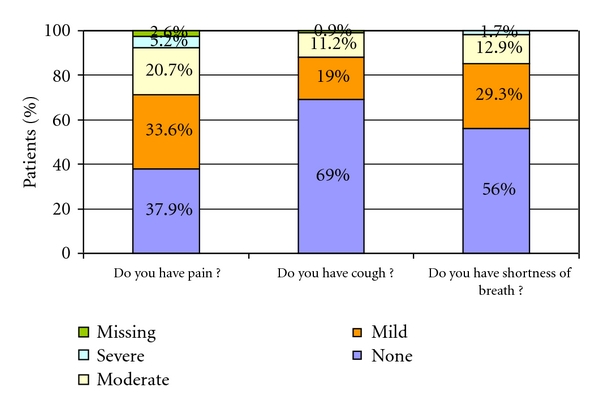
Cancer-related symptoms based on the 3-item questionnaire, all patients.

Figure 1 presents results from the 3-item Cancer-Related Symptoms Questionnaire for the pooled patient sample. The majority of patients had none to mild pain; however, 20.7% of patients reported moderate pain. 69% of patients had no cough, and the majority of patients had no shortness of breath (56%). Results were consistent across the mSTS and mBS subsamples.

### 3.4. Clinically Relevant Differences in Quality of Life and Utility

Table 6 shows the mean EQ-5D utility difference between health states (HS), and whether there is a minimal clinically relevant difference. The results indicate that patients on 1st-line chemotherapy (HS 1, *N* = 17, mean = 0.72) had a clinically relevant higher utility than patients on 2nd-line treatment (HS 2, *N* = 22, mean = 0.64). There was no clinically relevant difference in utility between patients on 1st-line (HS 1) and patients on 3rd-line treatment (HS 3; *N* = 12, mean = 0.77). Furthermore, patients on 2nd line treatment have clinically relevant lower utility than patients on 3rd line treatment. Comparing patients in progressive disease (HS 4, *N* = 28, mean = 0.56) against patients with stable disease that are off chemotherapy treatment (HS 5, *N* = 36, mean = 0.77) result in a clinically relevant lower utility for patients with progressive disease.

The analysis was repeated for the EORTC QLQ-C30 instrument. These results are presented in Table 6 below. In line with the results of the EQ-5D utility values, there is a decline in quality of life with patients on later lines of chemotherapy treatment. The change is small but clinically relevant, described in the table as “little change”, in quality of life between patients on 1st-(HS 1) and 2nd-(HS 2) line chemotherapy treatment. The change is also clinically relevant between patients on 1st-(HS 1) and patients on 3rd-(HS 3) line of chemotherapy treatment. But the change is not clinically relevant between patients on 2nd-(HS 2) and 3rd-(HS 3) line chemotherapy treatment. These results thus suggest that there is a clinically relevant change in quality of life between 1st-line treatment and 2nd/3rd-line treatment. For patients with progressive disease (HS 4), there was large decrease in quality of life compared to patients off chemotherapy with stable disease (HS 5).

### 3.5. Determinants of Quality of Life 

The regression model showed that the patient's health state (HS) and number of sites of metastases were statistically significant predictors of quality-of-life, as measured by the EORTC QLQ-C30 global health status score. Results are displayed in Table 7. The health state parameter estimate shows that, compared to patients in HS 5 (favourable response and off chemotherapy), patients in HS 1 (favourable response and on 1st-line chemotherapy) had an estimated −9.5 decrease on the EORTC QLQ-C30 quality of life scale. However, this effect is not statistically significant (*P* = 0.14). Patients in HS 2 and HS 3 (favourable response and on 2nd- and 3rd-line or higher chemotherapy, resp.) had a significantly decreased quality of life compared to HS 5, controlling for number of sites of metastases. The progressive disease health state (HS 4) had the most negative impact (−24.9) on overall quality of life when compared with HS 5. The results of the health state covariates are thus in line with what one would expect. The number of sites of metastases remained significant in the regression model. As expected, the analyses revealed that the number of sites of metastases had a negative impact on quality of life such that each additional site where metastasis was present would decrease quality of life by an estimated −10.1. 

## 4. Discussion 

This study presented utility estimates and HRQoL among a sample of patients with metastatic soft tissue and bone sarcoma, excluding GIST. The results indicated the significant negative impact on disease progression, as patients with progressive disease consistently reported lower quality of life, irrespective of which instrument and method was used. Overall, the results were similar in the mSTS and mBS subgroups. 

Patients on second-line treatment (HS 2) reported lower utility than patients on first-line treatment (HS 1). Contrary to what one would expect, patients in health state on 3rd-line or higher chemotherapy (HS 3) had higher utility than patients on 2nd-line chemotherapy (HS 2), although the difference is not statistically significant. There are several possible explanations for this. Patients that move on to receive higher lines of chemotherapy could potentially be more fit and in better health. Moreover, patients on higher lines of chemotherapy generally receive single-agent chemotherapies with less toxicity compared to multiagent chemotherapy regimens, such as doxorubicin plus ifosfamide, which are used in earlier lines of therapy. It should also be noted that the confidence intervals for HS 2 and HS 3 are overlapping as well as the lack of statistical significance comparing these health states, which makes it difficult to draw any clear conclusions. The uncertainty in these results is also reflected in the analysis of the VAS scores by health state, where the VAS score declines with higher lines of chemotherapy. Furthermore, comparing clinically relevant differences between HS 2 and HS 3, we see that there is a clinically relevant negative difference in utility but “no change” in the global QoL variable from the EORTC QLQ-C30 instrument. A larger patient sample could potentially provide more insight into the exact relationship, but the differences in results could also stem from a difference in capturing differences in quality of life between the EQ-5D and the EORTC QLQ-C30 questionnaires. 

After having run various specifications of the regression model, only the number of sites of metastases and health state remained significant. The regression model included a set of country indicator variables in the initial specification; however, these variables were removed, because the coefficients were not statistically significant, indicating that there were no differences in quality of life between countries. 

Comparing the EORTC QLQ-C30 scores reported in this study with a Swedish study presenting QLQ-C30 values in the general population indicates that mSTS and mBS patients have reduced overall quality of life as well as lower quality of life as measured on both the functional scale and the symptoms scale [17]. Comparative values for the general population, age, and sex standardized to the SABINE population, are displayed in Table 5. 

Disease symptoms are common and impacts on mSTS and mBS patients' quality of life. Pain is a significant symptom which a majority of patients (54.3%) experience as mild-to-moderate, and 5.2% of patients as severe in the 3-item Cancer-Related Symptoms Questionnaire. The reported pain score (27.6) on the EORTC QLQ-C30 questionnaire was also higher than pain in the general population (18.8 [17]). Respiratory symptoms are also common in mSTS/mBS patients. 42.2% of patients have mild-to-moderate shortness of breath. In addition, the dyspnoea score was relatively high (22.7) compared to that of the general population (17.4 [17]). Other disease symptoms that were drivers of the reduction of QoL were fatigue followed by insomnia. 

As sarcomas are a heterogeneous group of patients, only the most common soft tissue histological subtypes were included in this study (leiomyosarcoma, malignant fibrous sarcoma, liposarcoma, and synovial sarcoma). Thus, the prevalence of histological sub-types here is not directly comparable to population-based studies including all soft tissue sub-types. In addition, bone sarcoma patients were included; however, these results were presented separately due to the fact that QoL, and utility in these patients with so few patients is highly exploratory. 

The study has several strengths. The patient population is geographically diverse and from a large number of treatment centres dispersed throughout Europe and North America. Due to the rarity of the condition, the sample size of 120 patients was considered relatively large for a study of metastatic sarcoma (mSTS/mBS) treated with chemotherapy. The STS types included in this study have a world incidence rate ranging from 0.1 (synovial sarcoma) to 0.7 (liposarcoma) per 100,000 per year, while for BS, the annual incidence rate is 0.3 (Ewing's sarcoma) and 0.3 (osteosarcoma) [2]. In addition, the identification of health states which are associated with clinically meaningful differences in health-related quality of life provides quantification of the decrements associated with disease progression and use of chemotherapy which is important for more accurate health economic modelling. 

The limitations of this study include the possible bias in selecting healthier patients at the sites to participate in the study. Subsequent stratification of the analysis by health states (HS) further reduced the sample size within health states, especially for the mBS subsample. No information on chemotherapy drug regimen or adverse events was collected. Anthracycline-based regimens are limited by cumulative toxicity (e.g., doxorubicin, doxorubicin plus ifosfamide) while single-agent regimens have fewer toxicities (e.g., trabectedin and gemcitabine) but used for longer duration. Comparing different regimens on quality of life would be confounded by line of therapy, as drugs with cumulative toxicity are often used in early lines, while single-agent regimens with less toxicity are often used later. 

Among the few reported studies of quality-of-life in patients with metastatic sarcoma, none has presented utility values. Poveda et al. [11] assessed quality of life in patients participating in a phase II clinical study with the EORTC QLQ-C30 questionnaire. Quality of life did not seem to worsen during therapy. While Poveda et al. enrolled only patients participating in a clinical trial, our study enrolled patients in daily clinical practice indicating a better representative patient sample. In the absence of utility values from sarcoma patients, Soini et al. [18] used the EORTC QLQ-C30 results to predict the utility values by three generic quality of life measures (EQ-5D, 15D, and SF-6D) based on a published regression model [19]. The average expected utilities based on these instruments were 0.654, 0.736, and 0.668. Despite these differences, the predicted values are similar to the utility reported for the overall sarcoma sample from this study using the EQ-5D (0.69). 

Furthermore, the results of this study will fill the gap in the literature by providing health utility estimates for different sarcoma health states. As an example of the gap, an economic model submitted to the UK National Health Services (NHS) had to use utility values for lung cancer patients to substitute for sarcoma patients, due to lack of such data in sarcoma literature. The NHS appraisal committee questioned the appropriateness of using utility values from another condition, as a limitation of the submission. For accurate economic modelling and subsequent resource allocation decisions, it is critical to collect data on utility values for the mSTS/mBS population [20]. 

## 5. Conclusions 

This study demonstrated reduced quality of life in patients with mSTS and mBS who had a favourable response to chemotherapy. Quality of life and utility is especially low in patients with progressive disease. Highest quality of life and utility is experienced by patients who are off chemotherapy and in stable disease. Pain and respiratory symptoms are important common symptoms. The results from this study are appropriate for use in economic evaluations of treatments in metastatic soft tissue and bone sarcoma. 

## Figures and Tables

**Table 1:** Predefined health states.

Health state	Definition
1	1st-line chemotherapy, preprogressive disease
2	2nd-line chemotherapy, preprogressive disease
3	3rd-line or higher chemotherapy, preprogressive disease
4	Progressive disease (on or off chemotherapy)
5	After chemotherapy, pre progressive disease

**Table 2:** Patient characteristics.

	*N* = 116 (100%)
*Gender, N (%)*	

Female	68 (58.6%)
*Histology, N (%)*	
Soft tissue sarcoma	96 (82.8%)
Leiomyosarcoma	38 (32.8%)
Liposarcoma	23 (19.8%)
Synovial sarcoma	14 (12.1%)
Undifferentiated pleomorphic sarcoma/malignant fibrous histiocytoma	21 (18.1%)
Bone sarcoma	20 (17.2%)
Osteosarcoma	8 (6.9%)
Ewing's sarcoma	12 (10.3%)
*Site*, *N (%) *	
Extremity	41 (35.3%)
Retroperitoneum	19 (16.4%)
Superficial trunk and chest wall	5 (4.3%)
Head and neck	2 (1.7%)
Cutaneous	1 (0.9%)
Visceral non-GI	12 (10.3%)
Visceral GI	2 (1.7%)
Knee	1 (0.9%)
Hip	0
Shoulder	1 (0.9%)
Pelvis	4 (3.5%)
Femur/humerus	2 (1.8%)
Ribs	0
Other	24 (20.7%)
Missing	2 (1.7%)
*Site of metastases, N (%)*	
CNS	1 (0.6%)
Lung	74 (44.6%)
Liver	24 (14.5%)
Soft tissue	11 (6.6%)
Locoregional	28 (16.9%)
Other	25 (15.1%)
Missing	3 (1.8%)
*Receiving chemotherapy, N (%)*	
Yes	73 (62.9%)
*Number of sites of metastases*	
Mean (SD)	1.4 (0.7)
Minimum, maximum	1, 3
*Age at sarcoma diagnosis*	
Mean (SD)	47.7 (17.2)
Minimum, maximum	13.6, 83.7
*Age at metastatic disease diagnosis*	
Mean (SD)	49.5 (17.1)
Minimum, maximum	16.1, 83.8
*Age at attainment of current response status*	
Mean (SD)	51.6 (17)
Minimum, maximum	18.5, 84.3

**Table 3:** QoL based on the EQ-5D.

	Statistic	All (*N* = 116)	mSTS (*N* = 96)	mBS (*N* = 20)
*Mobility*	*N* (%)			
No problems		64 (55.2%)	52 (54.2%)	12 (60%)
Some problems		48 (41.4%)	40 (41.7%)	8 (40%)
Confined to bed		4 (3.5%)	4 (4.2%)	0
Missing		0	0	0
*Self-care*	*N* (%)			
No problems		95 (81.9%)	75 (78.1%)	20 (100%)
Some problems		20 (17.2%)	20 (20.8%)	0
Unable to wash or dress myself		0	0	0
Missing		1 (0.9%)	1 (1%)	0
*Usual activities*	*N* (%)			
No problems		48 (41.4%)	43 (44.8%)	5 (25%)
Some problems		60 (51.7%)	46 (47.9%)	14 (70%)
Unable to perform usual activities		7 (6%)	6 (6.3%)	1 (5%)
Missing		1 (0.9%)	1 (1%)	0
*Pain/discomfort*	*N* (%)			
No pain/discomfort		44 (37.9%)	38 (39.6%)	6 (30%)
Moderate pain/discomfort		66 (56.9%)	54 (56.3%)	12 (60%)
Extreme pain/discomfort		6 (5.2%)	4 (4.2%)	2 (10%)
Missing		0	0	0
*Anxiety/depression*	*N* (%)			
Not anxious/depressed		64 (55.2%)	53 (55.2%)	11 (55%)
Moderately anxious/depressed		49 (42.2%)	41 (42.7%)	8 (40%)
Extremely anxious/depressed		3 (2.6%)	2 (2.1%)	1 (5%)
Missing		0	0	0
*Utility score^1^*	*N*	114	94	20
	Mean (SD)	0.69 (0.26)	0.69 (0.26)	0.68 (0.27)
	Median	0.73	0.73	0.74
	Min, max	−0.08, 1	−0.08, 1	−0.08, 1
	95% CI	0.64, 0.73	0.64, 0.74	0.55, 0.8
*VAS score* ^2^	*N*	116	96	20
	Mean (SD)	64.97 (20.14)	65.75 (19.63)	61.25 (22.63)
	Median	70	70	65
	Min, max	0, 100	0, 100	15, 90
	95% CI	61.27, 68.68	61.77, 69.73	50.66, 71.84

Note: mSTS: metastatic soft tissue sarcoma, mBS: metastatic bone sarcoma; ^1^range −0.59 (worse than death)—1 (full health); ^2^range 0 (worst imaginable health)—100 (best imaginable health).

**Table 4:** EQ-5D utility values and VAS scores by health state.

	Statistic	EQ-5D utility^1^	VAS score^2^
*Health state *1	*N*	17	17
1st-line chemotherapy	Mean (SD)	0.72 (0.31)	68.82 (14.44)
	Median	0.76	70
	Min, max	−0.08, 1	30, 85
	95% CI	0.56, 0.88	61.4, 76.25
*Health state *2	*N*	22	23
2nd-line chemotherapy	Mean (SD)	0.64 (0.33)	65.04 (18.91)
	Median	0.73	70
	Min, max	−0.08, 1	15, 85
	95% CI	0.49, 0.78	56.86, 73.22
* Health state *3	*N*	12	12
3rd-line chemotherapy	Mean (SD)	0.77 (0.14)	63.75 (17.9)
	Median	0.75	64
	Min, max	0.59, 1	40, 90
	95% CI	0.68, 0.86	52.38, 75.12
*Health state *4	*N*	28	28
Progressive disease	Mean (SD)	0.56 (0.27)	50.79 (22.55)
	Median	0.66	50
	Min, max	0.08, 1	0, 100
	95% CI	0.46, 0.67	42.04, 59.53
*Health state *5	*N*	35	36
Off chemotherapy, preprogression	Mean (SD)	0.77 (0.14)	74.56 (16.03)
	Median	0.76	77
	Min, max	0.52, 1	39, 100
	95% CI	0.73, 0.82	69.13, 79.98

Note: ^1^Utility values range from −0.59 (worse than death)—1 (full health); ^2^VAS score range-from 0 to 100.

**Table 5:** Patients' QoL based on the QLQ-C30.

	Statistic	All	mSTS	mBS	General pop.^3^
*Global health status*					
QoL	Mean score (SD)	62.2 (22.8)	62.0 (22.5)	63.3 (24.5)	76.4, (21.8)
	Median	66.7	66.7	66.7	
	Min, max	16.7, 100	16.7, 100	25, 100	
	95% CI	58, 66.5	57.4, 66.6	51.8, 74.8	
*Functional scale* ^1^					
Physical functioning	Mean score (SD)	75.0 (19.8)	74.4 (20.3)	78.0 (17.0)	89.4, (17.2)
	SD				
	Min, max	26.7, 100	26.7, 100	46.7, 100	
	95% CI	71.3, 78.7	70.2, 78.5	70, 86	
Role functioning	Mean score (SD)	60.6 (33.6)	62.3 (32.9)	52.5 (36.4)	86.5, (24.4)
	Min, max	0, 100	0, 100	0, 100	
	95% CI	54.5, 66.8	55.7, 69	35.5, 69.5	
Emotional functioning	Mean score (SD)	72.6 (23.2)	72.6 (23.3)	72.9 (23.2)	81.2, (21.1)
	Min, max	0, 100	0, 100	8.3, 100	
	95% CI	68.4, 76.9	67.8, 77.3	62, 83.8	
Cognitive functioning	Mean score (SD)	83.2 (21.9)	84.2 (19.6)	78.3 (31.1)	88.4, (17.4)
	Min, Max	0, 100	0, 100	16.7, 100	
	95% CI	79.2, 87.2	80.2, 88.2	63.8, 92.9	
Social functioning	Mean score	65.9 (29.1)	65.8 (29.5)	66.7 (28.1)	90.8, (19.5)
	Min, max	0, 100	0, 100	0, 100	
	95% CI	60.6, 71.3	59.8, 71.8	53.5, 79.8	
*Symptoms scale^2^*					
Fatigue	Mean score (SD)	40.1 (28.4)	40.0 (28.5)	40.6 (28.2)	21, (21.7)
	Min, max	0, 100	0, 100	0, 100	
	95% CI	34.9, 45.3	34.3, 45.8	27.3, 53.8	
Nausea and vomiting	Mean score (SD)	13.8 (20.7)	14.6 (21.3)	10.0 (17.4)	3.3, (10.7)
	Min, max	0, 100	0, 100	0, 66.7	
	95% CI	10, 17.6	10.3, 18.9	1.8, 18.2	
Pain	Mean score (SD)	27.6 (27.2)	27.6 (27.3)	27.5 (27.2)	18.8, (25.5)
	Min, max	0, 100	0, 100	0, 83.3	
	95% CI	22.6, 32.6	22.1, 33.1	14.8, 40.2	
Dyspnoea	Mean score (SD)	22.7 (26.2)	22.6 (26.3)	23.3 (26.7)	17.4, (26)
	Min, max	0, 100	0, 100	0, 66.7	
	95% CI	17.9, 27.5	17.2, 27.9	10.8, 35.8	
Insomnia	Mean score (SD)	28.9 (29.9)	28.4 (29.7)	31.7 (31.5)	17.9, (26.3)
	Min, max	0, 100	0, 100	0, 100	
	95% CI	23.4, 34.5	22.3, 34.5	16.9, 46.4	
Appetite loss	Mean score (SD)	19.8 (27.1)	21.2 (28.7)	13.3 (16.8)	4.3, (14.7)
	Min, max	0, 100	0, 100	0, 33.3	
	95% CI	14.8, 24.8	15.4, 27	5.5, 21.2	
Constipation	Mean score (SD)	24.7 (30.5)	26.4 (31.3)	16.7 (25.4)	5.2, (15.7)
	Min, max	0, 100	0, 100	0, 66.7	
	95% CI	19.1, 30.3	20, 32.7	4.8, 28.5	
Diarrhea	Mean score (SD)	11.8 (22.9)	11.1 (20.3)	15.0 (33.3)	5.2, (15.4)
	Min, max	0, 100	0, 66.7	0, 100	
	95% CI	7.6, 16	7, 15.2	−0.6, 30.6	
Financial difficulties	Mean score (SD)	26.1 (32.4)	25.3 (32.5)	30.0 (32.3)	7, (19.7)
	Min, max	0, 100	0, 100	0, 100	
	95% CI	20.1, 32.1	18.6, 31.9	14.9, 45.1	

Note: The following scales had missing values: Global QoL, physical functioning, Insomnia, and financial difficulties ^1^a higher score indicates better quality of life ^2^a higher score indicates more symptoms; ^3^General population values, age and sex adjusted for SABINE population [17].

**Table 6:** Clinically relevant differences between health states.

Health state comparisons	EQ-5D (mean utility)		QLQ-C30 (mean global QoL)	
	Difference	Clinically relevant difference^1^	Difference	Clinically relevant difference^2^
1st-line CT (HS 1) versus 2nd-line CT (HS 2)	0.08	Positive	7.46	Little change
1st-line CT (HS 1) versus 3rd-line CT (HS 3)	−0.05	None	7.84	Little change
2nd-line CT (HS 2) versus 3rd-line CT (HS 3)	−0.14	Negative	0.38	No change
Progressive disease (HS 4) versus Off CT, preprogression (HS 5)	−0.21	Negative	−30.26	Very much change

Note: ^1^0.074 difference in mean utility, ^2^“little change” (5–10 points change in the score), a “moderate change” (10–20 points), and “very much change” (>20 points).

**Table 7:** Multiple linear regression analysis, with health-related quality of life as dependent variable.

Parameter	Estimate	Standard error	*t* value	Pr > |*t*|
Intercept	90.41	5.95	15.20	<.0001
Health State 1	−9.48	6.29	−1.51	0.1353
Health State 2	−15.92	5.97	−2.67	0.0092
Health State 3	−19.97	7.00	−2.85	0.0054
Health State 4	−24.85	5.68	−4.38	<.0001
Health State 5	0.00	NA	NA	NA
Number of sites of metastases	**−10.10**	**3.13**	−3.23	0.0018

Note: Refer to Table 1 for a definition of the health states. Health state 5 is the reference group.
